# m6A Regulator-Mediated RNA Methylation Modification Patterns are Involved in the Pathogenesis and Immune Microenvironment of Depression

**DOI:** 10.3389/fgene.2022.865695

**Published:** 2022-04-11

**Authors:** Ye Wang, Xinyi Wang, Chenyi Yang, Wei Hua, Haiyun Wang

**Affiliations:** ^1^ The Third Central Clinical College of Tianjin Medical University, Tianjin, China; ^2^ Tianjin Key Laboratory of Extracorporeal Life Support for Critical Diseases, Tianjin, China; ^3^ Artificial Cell Engineering Technology Research Center, Tianjin, China; ^4^ Tianjin Institute of Hepatobiliary Disease, Tianjin, China; ^5^ Nankai University Affinity the Third Central Hospital, Tianjin, China

**Keywords:** depression, immune microenvironment, N6-methyladenosine, biomarker, epigenetics

## Abstract

Depression is a genetical disease characterized by neuroinflammatory symptoms and is difficult to diagnose and treat effectively. Recently, modification of N6-methyladenosine (m6A) at the gene level was shown to be closely related to immune regulation. This study was conducted to explore the effect of m6A modifications on the occurrence of depression and composition of the immune microenvironment. We downloaded gene expression profile data of healthy and depressed rats from the Gene Expression Omnibus. We described the overall expression of m6A regulators in animal models of depression and constructed risk and clinical prediction models using training and validation sets. Bioinformatics analysis was performed using gene ontology functions, gene set enrichment analysis, gene set variation analysis, weighted gene co-expression network analysis, and protein-protein interaction networks. We used CIBERSORT to identify immune-infiltrating cells in depression and perform correlation analysis. We then constructed two molecular subtypes of depression and assessed the correlation between the key genes and molecular subtypes. Through differential gene analysis of m6A regulators in depressed rats, we identified seven m6A regulators that were significantly upregulated in depressed rats and successfully constructed a clinical prediction model. Gene Ontology functional annotation showed that the m6A regulators enriched differentially expressed genes in biological processes, such as the regulation of mRNA metabolic processes. Further, 12 hub genes were selected from the protein-protein interaction network. Immune cell infiltration analysis showed that levels of inflammatory cells, such as CD4 T cells, were significantly increased in depressed rats and were significantly correlated with the depression hub genes. Depression was divided into two subtypes, and the correlation between hub genes and these two subtypes was clarified. We described the effect of m6A modification on the pathogenesis of depression, focusing on the role of inflammatory infiltration.

## Introduction

Depression is a common mental illness that is classified as having a global disease burden and is the leading cause of suicide (Hasin et al., 2018). According to the World Health Organization, 5.0% of adults and 5.7% of adults over the age of 60 years suffer from depression (World Health Organization, 2021). Depression is typically diagnosed by psychiatrists based on subjective identification of symptom clusters, resulting in a high rate of misdiagnosis (Pan et al., 2018) and suboptimal treatment strategies (Chen et al., 2008).

At present, many biomarkers, such as thalamic-pituitary-adrenal axis-related hormones and metabolic indexes, have little practical clinical applications because of their limited sensitivity and specificity (Strawbridge et al., 2017). Woo et al. (2018) examined gene expression in peripheral blood samples from 38 patients with depression and 14 healthy controls and identified seven differentially expressed genes between the two groups. Further, Long et al. (2021) screened three candidate genes with high auxiliary value in depression diagnosis by analyzing two datasets (GSE53987 and GSE98793) in a comprehensive database of gene expression. However, most of these studies only identified differentially expressed genes but did not further explore their potential biomolecular mechanisms. Detailed studies of pathogenesis of depression are needed to help clarify its diagnosis and treatment. A recent study showed that children of depressed parents are three times more likely to suffer from severe depression than those of non-depressed parents, suggesting the genetic susceptibility of patients to depression (Weissman et al., 2016). Increasing studies have also indicated that neuroinflammation increases the risk of depression or changes the trajectory of depression (Benatti et al., 2016).

Within the emerging field of post-transcriptional gene regulation research, the N6-methyladenosine (m6A) modification of mRNA, which widely occurs in eukaryotes, has attracted attention because of its critical roles in various biological processes (Shi et al., 2019; He and He, 2021). The m6A modification exhibits dynamic and reversible characteristics, including as a “writer” (methyltransferase), “eraser” (demethylase), and “reader” (methyl binding protein) (Yang et al., 2018). Many m6A regulators are key factors in RNA methylation, in stability, translation, degradation, transport, and splicing (Han et al., 2021). Other studies showed that m6A is associated with nervous system development and the pathology of neurodegenerative diseases (Widagdo et al., 2016; Li et al., 2018). For example, the expression of the obesity-related gene FTO (an RNA demethylase) is downregulated in the hippocampus of in a mouse model of depression, whereas overexpression of FTO has antidepressant effects (Liu et al., 2021).

Researchers have shown that regulation of the CaMKII/CREB signaling pathway involved in m6A plays a key role in hippocampal synaptic plasticity, thus improving depression-like symptoms induced by chronic restraint stress (Shen et al., 2021). Huang et al. (2020) explored the functional relationship between circSTAG1 and m6A methylation in depression models; their findings suggested that circSTAG1 is a new therapeutic target for treating depression. Further evidence confirmed that m6A is involved in innate and acquired immune responses (Zheng et al., 2017). For example, the cytokine signal transduction inhibitor protein family is strongly affected by m6A modification (Li H.-B. et al., 2017) and plays an important role in T cell proliferation and differentiation (Palmer and Restifo, 2009). Previous studies suggested a fundamental role for m6A modifications in the tumor immune microenvironment (Han et al., 2019; Zhang et al., 2020). More recent studies revealed that m6A plays a similarly crucial role in the immune microenvironment of periodontitis (Zhang et al., 2021) and severe asthma (Sun et al., 2021). For multifaceted diseases such as depression, which is influenced by multiple factors such as genetic susceptibility and the inflammatory response, early diagnosis and treatment are critical.

In this study, we systematically explored the relationship between m6A and the pathogenesis and immune infiltration of depression using bioinformatics analysis, including differential expression, prediction model construction, functional enrichment analysis, protein-protein interaction (PPI), and other methods, to identify biomarkers and potential therapeutic applications.

## Materials and Methods

### Data Sources

**FIGURE 1:**
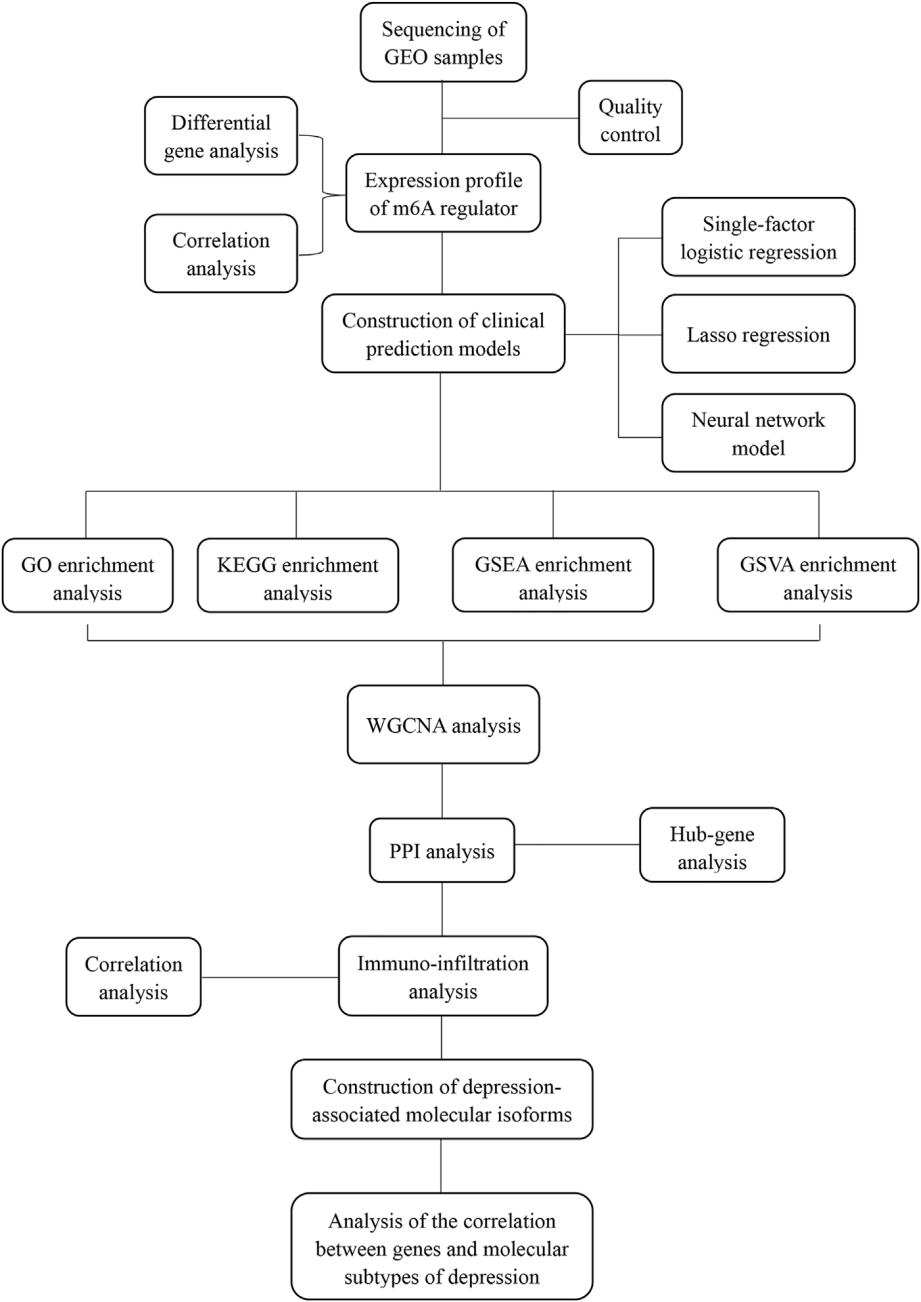
Analysis flow chart.

Gene expression profile data of the animal models of depression were downloaded from the GEO database (https://www.ncbi.nlm.nih.gov/geo/). The first dataset GSE63377 (Yamamoto et al., 2015), which was obtained from the GPL15084 sequencing platform, included two cases of depression-associated inflammation in the cerebellum and prefrontal cortex and two cases of depression with normal tissue. The second dataset, GSE124387 (Zheng et al., 2019), which was obtained from the GPL1355 sequencing platform, included three cases of depression characterized by inflammation in the postfrontal cortex and three cases with normal tissue. The third dataset, GSE86392 (Wang et al., 2017), which was obtained from the GPL20084 sequencing platform, included three cases of depression characterized by inflammation of the hippocampus, frontal cortex, and pituitary tissue and three cases that exhibited normal tissue after recovery from depression (Figure 1).

### Data Preprocessing

To increase the breadth and accuracy of the analysis, the datasets GSE63377 and GSE124387 were merged into the training set and the dataset GSE86392 was used as the validation set. The R package edgeR (Robinson et al., 2010) was used to remove batch effects and perform background correction for these three datasets. The correction effect was visualized using the R package ggplot2 (Villanueva and Chen, 2019). The intersection of the GSE63377 and GSE124387 gene datasets was used to generate the training set.

### Expression Profiling of m6A Methylation Regulators

To analyze the expression of m6A regulator expression in depression, the R package RCircos was used to analyze the chromosomal location of the m6A regulator. Next, the *p*-values of m6A-related genes in the experimental and control groups were determined using Wilcoxon test and visualized in the ggplot2 package. Differentially expressed genes of m6A methylation regulators were analyzed using the R package limma (Robinson et al., 2010) and visualized using the R package EnhancedVolcano. To analyze the correlation and interaction of the m6A regulators, the Spearman algorithm was used to perform correlation analysis of the m6A regulator, which was visualized using the R package ggcorrplot.

### Risk Model and Clinical Prediction Model Construction

To analyze the expression of m6A regulators in depression, we first identified key genes associated with depression using single-factor logistic regression analysis of the training set and then used the least absolute shrinkage and selection operator (LASSO) algorithm for dimensionality reduction analysis and to validate key genes associated with depression. Subsequently, depression-related eigengenes were incorporated into the model, and the R package keras (cite) was used to build a back-propagation neural network model of the classifier architecture. The neural network model includes six layers, namely: input (activation = “relu”), Dense, Dropout (rate = 0.5), Dense, Dropout (rate = 0.5), Output (activation = “sigmoid”), combined with the optimizer, and a custom penalty coefficient. To quantify the discriminative performance of the model, a receiver operating characteristic curve was generated by comparing the predicted values of the neural network with the observed values to evaluate the performance of the clinical prediction model.

### m6A Regulator Functional Enrichment Analysis

Gene ontology (GO) analysis is commonly used for large-scale functional enrichment studies, including for evaluation of biological processes, molecular functions, and cellular components (Ashburner et al., 2000). Kyoto Encyclopedia of Genes and Genomes (KEGG) is a widely used database for storing information on genomes, biological pathways, diseases, and drugs (Kanehisa and Goto, 2000). GO annotation and KEGG pathway enrichment analyses of m6A-related genes were performed using the clusterProfiler package of R (Yu et al., 2012); a false discovery rate of <0.05 was considered as the cutoff for determining statistically significant results.

### Gene Set Enrichment Analysis and Gene Set Variation Analysis

To investigate the differences in biological processes between the different groups, gene set enrichment analysis (GSEA) based on the gene expression profiling dataset of depression in the *Rattus norvegicus* model. GSEA is a computational method used to analyze whether a specific gene set is significantly different between two biological states (Subramanian et al., 2005) and is often used to estimate changes in pathways and biological process activity in samples of expression datasets. Gene set variation analysis (GSVA) is a GSEA algorithm. Unsupervised classification of samples can also be performed based on changes in pathway activity, gene expression levels, and multiple pathway information. We used the R package clusterProfiler and species parameter selection, org. Rn.eg.db, for GSEA. A *p*-value < 0.05 was considered to indicate statistically significant results. The R package GSVA (Hänzelmann et al., 2013) was used to perform GSVA, and the difference in the related GSVA pathway between the experimental and control groups was determined using linear fitting and a Bayesian network algorithm. Statistical significance was set at *p* < 0.05.

### Weighted Gene Co-Expression Network Analysis

To analyze the signature gene set of depression, weighted gene co-expression network analysis (WGCNA) was performed using the R package WGCNA (Langfelder and Horvath, 2008). First, hierarchical clustering was performed on the gene expression data of the cleaned training to remove outliers. The best soft threshold was chosen based on the R^2^ and slope values, after which the scale-free network was validated. The adjacency matrix and topological matrix were determined and dissimilarity analyses were carried out, after which the network module was identified by dynamic shear tree analysis. Finally, correlation analysis was carried out in combination with depression phenotype information.

### Construction of Protein-Protein Interaction Network

The PPI network is composed of individual proteins that interact with each other to participate in all aspects of life processes, such as biological signal transmission, gene expression regulation, energy and material metabolism, and cell cycle regulation. Systematic analysis of the interaction of a large number of proteins in biological systems is useful for understanding the working principle of proteins in biological systems, response mechanism of biological signals and energy metabolism under special physiological conditions such as diseases, and functional connections between proteins. The STRING database (Szklarczyk et al., 2019) is used to search for interactions between known and predicted proteins. We used the STRING database to construct a PPI network of m6A-related genes and Cytoscape (v3.8.2) (Shannon et al., 2003) to visualize the PPI network model.

### Identification and Correlation Analysis of Immune Infiltration in Depression

The immune microenvironment is mainly composed of immune cells, inflammatory cells, fibroblasts, interstitial tissue, and various cytokines and chemokines that are loaded into comprehensive systems. Analysis of immune cell infiltration in tissues is important in disease research and for predicting treatments and prognosis. CIBERSORT is a deconvolution algorithm used to analyze the expression matrix of immune cell subtypes based on the principle of linear support vector regression. CIBERSORT uses RNA-sequencing data to estimate the abundance of immune cells in tissues (Newman et al., 2019). We used the CIBERSORT algorithm to estimate the abundance of 24 types of immune cells in high- and low-risk samples in the dataset, and the immune cell composition of disease samples and normal samples were visualized using boxplots. All analyses and visualizations were carried out in R software. Differences in the proportion of immune cells between the diseased and normal sample groups were calculated using the Wilcoxon test, and a *p*-value < 0.05 was considered to indicate statistically significant results.

Based on the results of immune infiltration analysis, the R package corrr was used to calculate the correlation between key genes and immune signatures to clarify the relationship between the key genes of depression and immune characteristics of species. The correlation matrix was visualized using the R package ggcorrplot.

### Construction of the Depression-Related Molecular Subtype

To analyze the subtypes of depression-related molecules, the training set raw gene expression data were used; the raw data was normalized using LASSO regression. Next, the R package ConsensusClusterPlus (Wilkerson and Hayes, 2010) was used to perform consistent clustering analysis using the PAM algorithm, and the optimal number of clusters was determined by principal coordinate analysis. Finally, the R packages pheatmap and Rtsne were used for visualization.

### Correlation Analysis of Key Genes and Depression-Related Molecular Subtypes

Based on the analysis results of depression-related molecular subtypes, the correlation between key genes and depression-related molecules was calculated using Spearman correlation analysis, and the results were visualized using the R package ggcorrplot.

### Statistical Analysis

All data processing and analyses were performed using R software (version 4.1.2). To compare two groups of continuous variables, the statistical significance of normally distributed variables was estimated using the independent Student *t*-test, and differences between non-normally distributed variables were analyzed using Mann-Whitney *U* test (Wilcoxon rank sum test). The chi-square test or Fisher’s exact test was used to compare and analyze statistical significance between the two groups of categorical variables. Correlation coefficients between different genes were calculated using Spearman correlation analysis. All statistical *p*-values were two-sided, and *p* < 0.05 was considered to indicate statistically significant results.

## Results

### Gene Chip Quality Control

**FIGURE 2:**
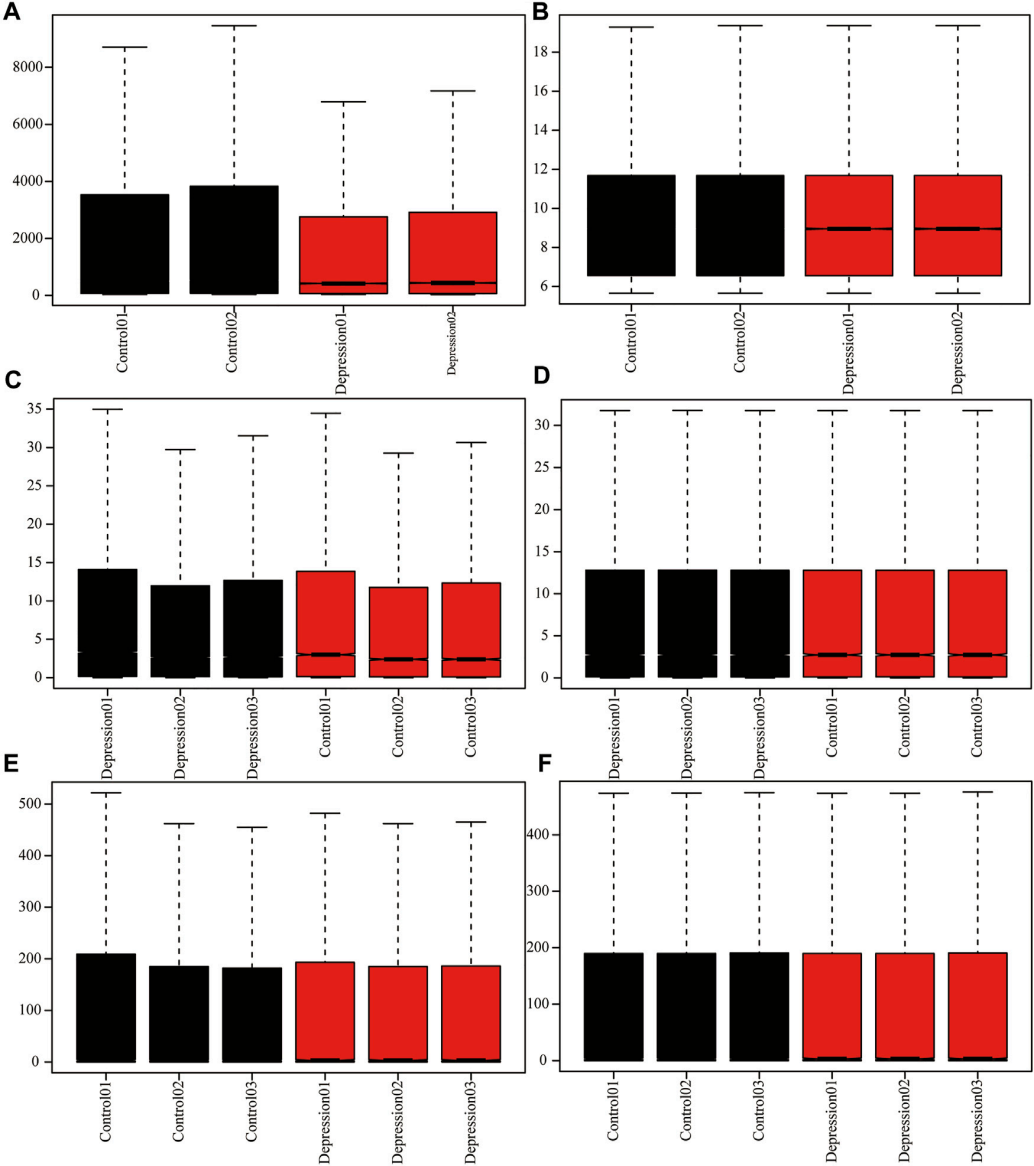
Adjusted for depression data. **(A,B)**: GSE63377 chip data before and after background correction. **(C,D)**: GSE124387 chip data before and after correction. **(E,F)**: GSE86392 gene chip data before and after correction.

To analyze the overall expression of m6A methylation regulators in animal models of depression, we comprehensively analyzed expression in depressed and normal tissues from GEO data using the de-batch effect and background correction (Figure 2).

### Expression Profile of m6A Regulators in Depression

**FIGURE 3:**
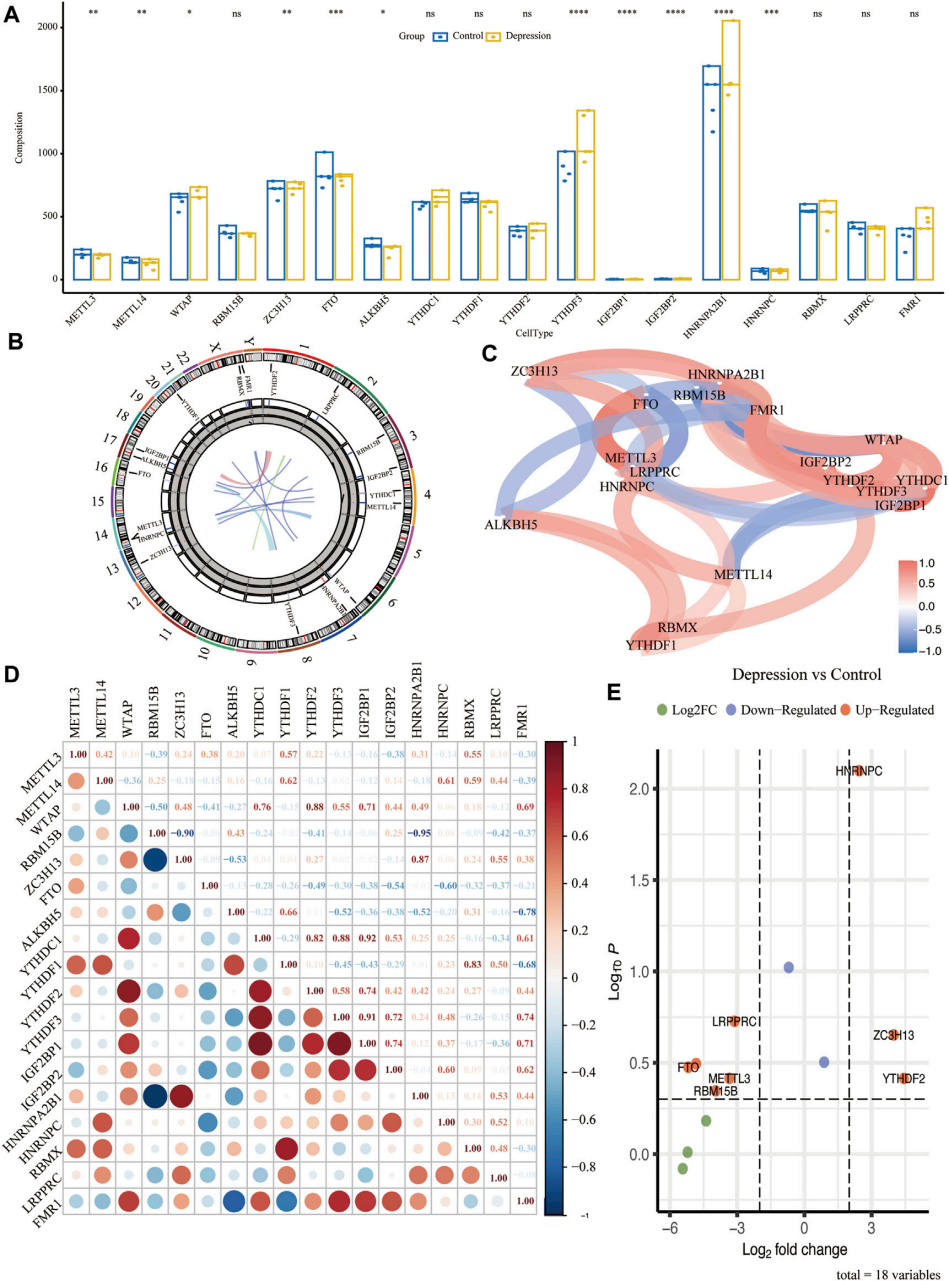
Overall expression of m6A methylation regulators in animal models of depression. **(A)** Difference in expression of the m6A regulator between the experimental and control groups. **(B)** Chromosome localization map of m6A regulators. **(C)** Correlation network diagram of m6A regulators. **(D)** Correlation heat map of m6A regulators. **(E)** Volcano plot of the results of the differential genetic analysis of m6A regulators.

To analyze the overall expression of m6A regulators in animal models of depression, we analyzed their chromosomal localization (Figure 3B). Next, we calculated the *p*-values of the m6A-related genes in the experimental and control groups using the Wilcoxon test. The results showed that *METTLE3*, *METTLE14*, *YTHDF3*, *IGF2BP1*, and seven other genes were significantly differentially expressed (*p* < 0.05) (Figure 3A). Next, we performed differential gene analysis of m6A regulators in depression and found that *FTO*, *RBM15B*, *METTL3*, *LRPPRC*, *HNRNPC*, *ZC3H13*, and *YTHDF2* were significantly upregulated in depressed rats (Figure 3E). The correlation and interaction of the m6A regulators were analyzed using the Spearman algorithm (Figure 3C). Genes with an absolute value of correlation coefficient ≥0.7 were considered as significantly correlated (Figure 3D).

### Construction of Clinical Prediction Models

**FIGURE 4:**
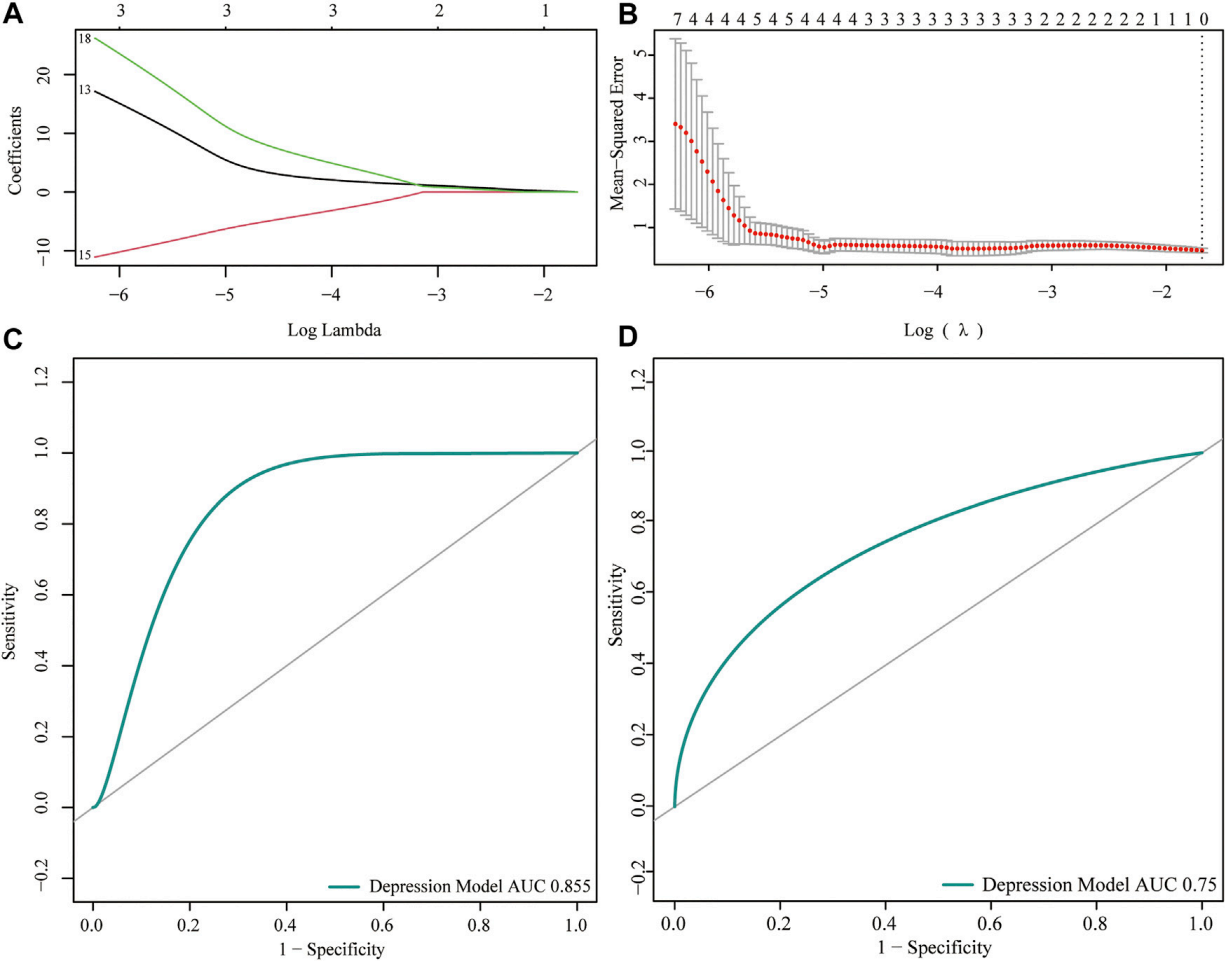
Predictive power analysis of neural network models for depression. **(A,B)** LASSO regression analysis identified key genes for m6A regulators. **(C)** Receiver operating characteristic (ROC) curve of the neural network model test set; the abscissa represents the specificity and ordinate represents the sensitivity. **(D)** ROC curve of the validation set of the neural network model; the abscissa represents the specificity and ordinate represents the sensitivity.

To analyze the expression of the m6A regulator in depression, we first performed training set univariate logistic regression analysis to identify key genes related to depression, and then conducted LASSO algorithm analysis to perform dimensionality reduction analysis and verify the key genes related to depression (Figures 4A,B). The depression-related eigengenes were incorporated into the model; the back-propagation neural network model of the classifier architecture was constructed, and the area under the test set receiver operating characteristic curve was 0.855 (Figure 4C). Next, a receiver operating characteristic curve was generated for the validation set by comparing the predicted values of the neural network with the actual depression observed in the validation set (Figure 4D).

### Functional Enrichment Analysis

**FIGURE 5:**
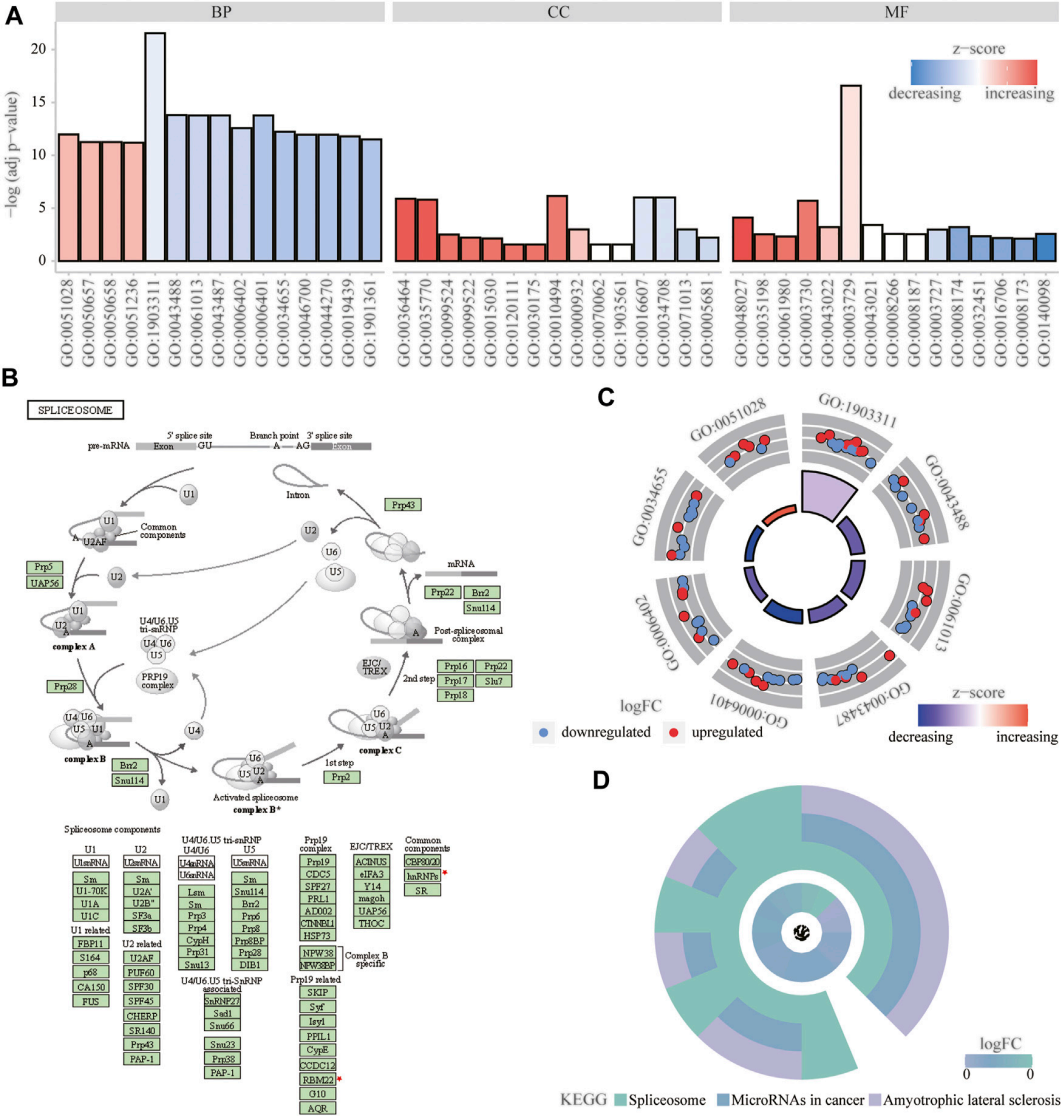
Functional enrichment analysis of m6A regulator. **(A)** First 15 items enriched for biological processes, molecular functions, and cellular components the abscissa represents the GO term and ordinate is the -log (adj *p*-value). Band colors: blue represents downregulation and red represents upregulation. **(B)** Significantly enriched KEGG pathway; rno03040: spliceosome. **(C)** m6Aregulation of the first eight items of biological processes; red represents upregulation, and blue represents downregulation. **(D)** KEGG enrichment results; the outer circle represents the KEGG pathway and inner circle represents the log fold-change size.

We analyzed the effect of the m6A regulators on the biologically relevant functions of mice in the experimental and control groups (Table 1). The results of GO function annotation of the m6A regulators showed that the differentially expressed genes were mainly enriched in biological processes, such as regulation of mRNA metabolic process, regulation of mRNA stability, regulation of mRNA catabolic process, regulation of RNA stability, RNA catabolic process, mRNA binding, mRNA 3′-untranslated region binding, mRNA 5′-untranslated region binding, ribonucleoprotein complex binding, and ribosome binding, as well as in the production of cytoplasmic stress granules, methyltransferase complexes, nuclear specks, cytoplasmic ribonucleoprotein granules, and ribonucleoprotein granules (Figure 5A). We analyzed the regulation of the m6A regulators in the first eight enrichment results of the resulting GO biological processes (Figure 5C). Simultaneously, these m6A-related regulators were enriched in the spliceosome KEGG pathway (Table 2), and we examined the enrichment of the expression levels of m6A-related regulators in the pathway rno03040 in detail (Figures 5B,D).

**TABLE 1:** GO enrichment analysis (TOP5).

Category	ID	Description	*p* Value
BP	GO:1903311	Regulation of mRNA metabolic process	5.99E-25
BP	GO:0043488	Regulation of mRNA stability	6.53E-17
BP	GO:0061013	Regulation of mRNA catabolic process	1.10E-16
BP	GO:0043487	Regulation of RNA stability	1.50E-16
BP	GO:0006401	RNA catabolic process	1.81E-16
MF	GO:0003729	mRNA binding	4.21E-19
MF	GO:0003730	mRNA 3′-UTR binding	6.51E-08
MF	GO:0048027	mRNA 5′-UTR binding	3.89E-06
MF	GO:0043021	Ribonucleoprotein complex binding	2.56E-05
MF	GO:0043022	Ribosome binding	5.67E-05
CC	GO:0010494	Cytoplasmic stress granule	9.10E-09
CC	GO:0034708	Methyltransferase complex	2.82E-08
CC	GO:0016607	Nuclear speck	3.75E-08
CC	GO:0036464	Cytoplasmic ribonucleoprotein granule	6.38E-08
CC	GO:0035770	Ribonucleoprotein granule	1.02E-07

**TABLE 2:** KEGG enrichment analysis.

Category	ID	Description	*p* Value
KEGG	rno03040	Spliceosome	1.32E-03
KEGG	rno05206	MicroRNAs in cancer	1.25E-01
KEGG	rno05014	Amyotrophic lateral sclerosis	1.56E-01

### Gene Set Enrichment Analysis and Gene Set Variation Analysis

**FIGURE 6:**
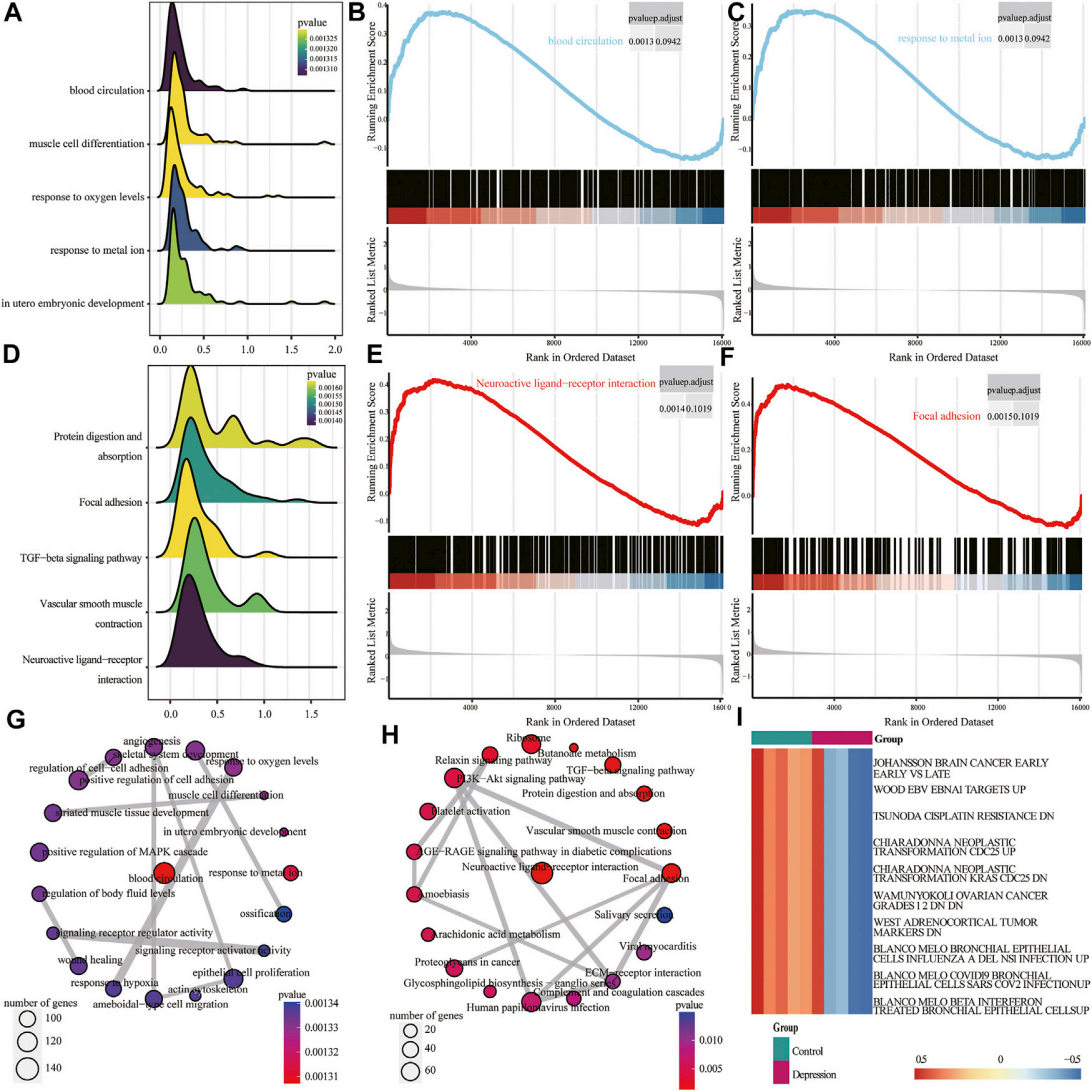
Gene set enrichment analysis (GSEA) and gene set variation analysis (GSVA) of experimental and control groups. **(A)** Mountain map of the first five items of the GSEA Gene Ontology (GO) analysis results; the abscissa is normalized enrichment score (NES), ordinate shows the GO terms, size of the mountain represents the number of genes, and color represents the *p*-value. **(B,C)** Clustering of the first two items in GSEA GO. **(D)** Mountain map of the first five items of the GSEA KEGG results; the abscissa is NES, ordinate shows the KEGG terms, size of the mountain represents the number of genes, and color represents the *p*-value. **(E,F)** GSEA KEGG of clustering of the first two items. **(G)** Interaction network diagram of GSEA GO. **(H)** Interaction network diagram of GSEA KEGG. **(I)** Heat map of differential expression of GSVA; red represents upregulation and blue represents downregulation.

Next, we performed GSEA of all genes between the experimental and control groups (Table 3). The results showed that the following biological processes differed between the control and experimental groups: blood circulation, response to metal ions, *in utero* embryonic development, muscle cell differentiation, and response to oxygen levels (Figure 6A). The first two enrichment results of the biological process were analyzed (Figures 6B,C). We further identified significant differences in neuroactive ligand-receptor interaction, focal adhesion, vascular smooth muscle contraction, protein digestion and absorption, and the TGF-β signaling pathway (Figure 6D). The top two results of pathway enrichment were subjected to further enrichment analysis (Figures 6E,F). Next, we conducted further interaction analysis of the GO and KEGG enrichment results from GSEA (Figures 6G,H). To clarify the specific mechanism of depression, we performed a GSVA of all genes (Table 4). The results revealed significant differences in biological processes such as Blanco melo COVID-19 bronchial epithelial cells SARS-CoV-2 infection up, Blanco melobronchial epithelial cells influenza a del ns1 infection up, West adrenocortical tumor markers, and Wamunyokoli ovarian cancer grades 12 (Figure 6I).

**TABLE 3:** GSEA enrichment analysis.

Category	ID	ES	NES	*p* Value
GSEA GO enrichment analysis				
BP	Blood circulation	0.37661709	1.54144753	0.00130719
BP	Response to metal ion	0.35000789	1.43162905	0.00131406
BP	*In utero* embryonic development	0.33371112	1.35995142	0.00132626
BP	Muscle cell differentiation	0.36618632	1.47293194	0.00132979
BP	Response to oxygen levels	0.36120132	1.46810233	0.00132979
MF	Signaling receptor regulator activity	0.34917598	1.41486991	0.00133511
MF	Signaling receptor activator activity	0.36242612	1.46393421	0.00133869
MF	Receptor ligand activity	0.36892487	1.48774541	0.00134228
MF	Actin binding	0.36858646	1.47337515	0.00136612
MF	Cell adhesion molecule binding	0.44053979	1.70847133	0.001443
CC	Actin cytoskeleton	0.34962783	1.41170259	0.0013369
CC	Receptor complex	0.37117819	1.48901146	0.0013459
CC	External encapsulating structure	0.5368778	2.15390376	0.00134771
CC	Extracellular matrix	0.53569273	2.14740749	0.00135318
CC	Contractile fiber	0.41358761	1.58654453	0.00143885
GSEA KEGG enrichment analysis				
KEGG	Neuroactive ligand-receptor interaction	0.41074986	1.62854009	0.00137741
KEGG	Focal adhesion	0.485989	1.79470041	0.00152439
KEGG	Vascular smooth muscle contraction	0.49803859	1.76525	0.0015748
KEGG	Protein digestion and absorption	0.57855845	1.96048059	0.00163132
KEGG	TGF-beta signaling pathway	0.52845983	1.76961378	0.00164474

**TABLE 4:** GSVA enrichment analysis.

ID	LogFC	*p* Value	adj.*p* Value
Blanco melo COVID19 bronchial epithelial cells SARS COV 2 infection up	−1.133532	0.01738955	0.21481928
Blanco melo beta interferon treated bronchial epithelial cells up	−1.133532	0.01738955	0.21481928
Blanco melo bronchial epithelial cells influenza a del NS1 infection up	−1.133532	0.01738955	0.21481928
West adrenocortical tumor markers DN	−1.133532	0.01738955	0.21481928
Wamunyokoli ovarian cancer grades 1 2 DN	−1.133532	0.01738955	0.21481928
Chiaradonna neoplastic transformation KRAS CDC25 DN	−1.133532	0.01738955	0.21481928
Chiaradonna neoplastic transformation CDC25 up	−1.133532	0.01738955	0.21481928
TSUNODA cisplatin resistance DN	−1.133532	0.01738955	0.21481928
Wood EBV EBNA1 targets up	−1.133532	0.01738955	0.21481928
Johansson brain cancer early vs. late DN	−1.133532	0.01738955	0.21481928

### Weighted Gene Co-Expression Network Analysis

**FIGURE 7:**
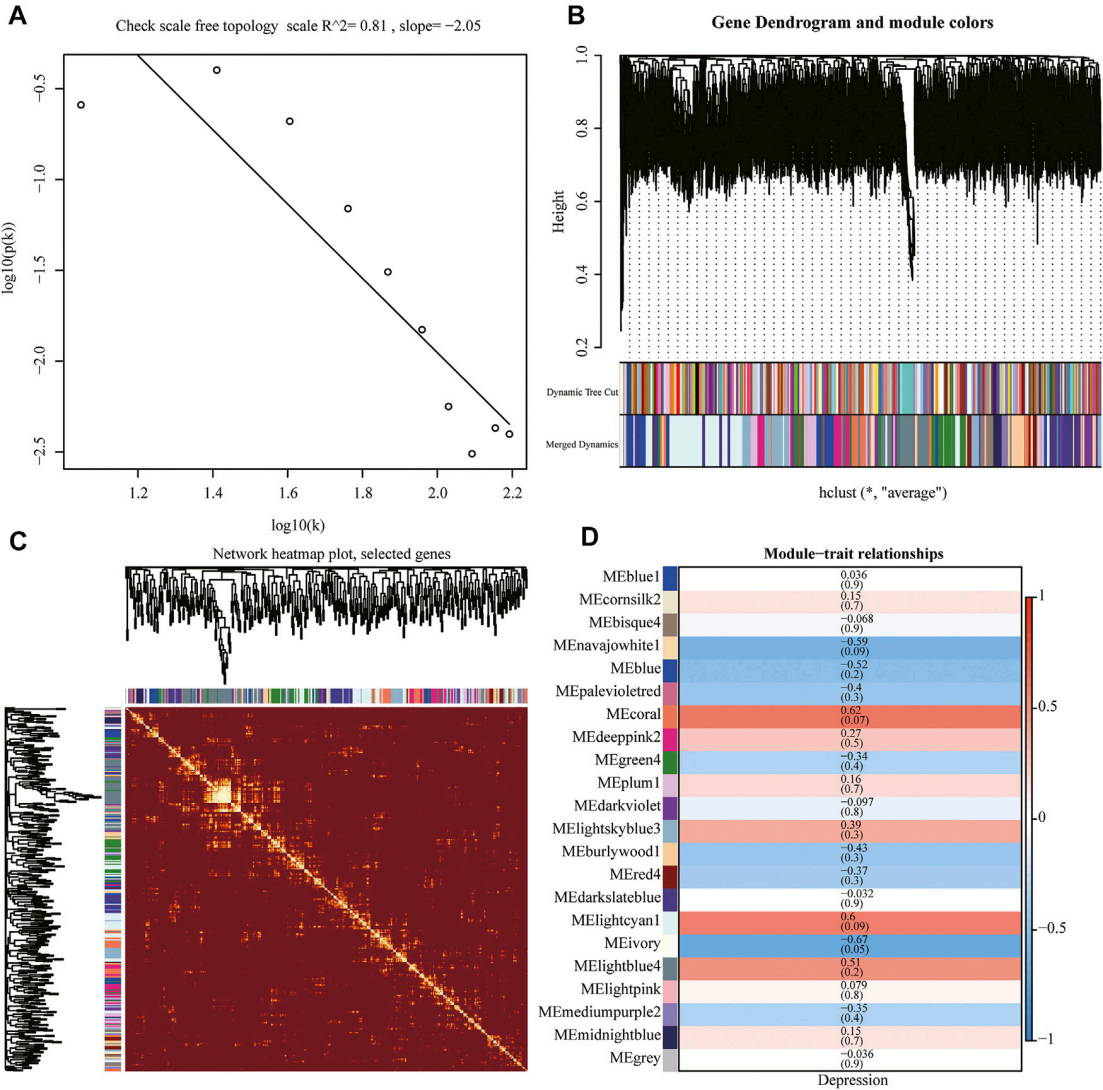
Weighted gene co-expression network analysis (WGCNA). **(A)** Scale-free network verification graph (R^2^ > 0.8, slope < 0), conforming to the scale-free network standard. **(B)** Dynamic clipping tree clustering diagram; the abscissa is the clustering module and ordinate is the tree height. **(C)** TOM network clustering heatmap. **(D)** Heat map of correlations between WGCNA network modules and depression.

We performed WGCNA using the whole genome of the training set. First, we performed a hierarchical clustering analysis of the samples, removed outlier samples, and identified network modules using dynamic clipping trees (Figure 7B). After selecting the optimal soft threshold, the scale-free network was verified, and the results showed that the scale-free network was successfully established (R^2^ = 0.8, slope < 0) (Figures 7A,C). Next, we performed correlation analysis using a scale-free network module and an external module (depression), which revealed a significant association between the ivory module and depression (Figure 7D).

### m6A Regulator Protein-Protein Interaction Network Between Experimental and Control Groups

**FIGURE 8:**
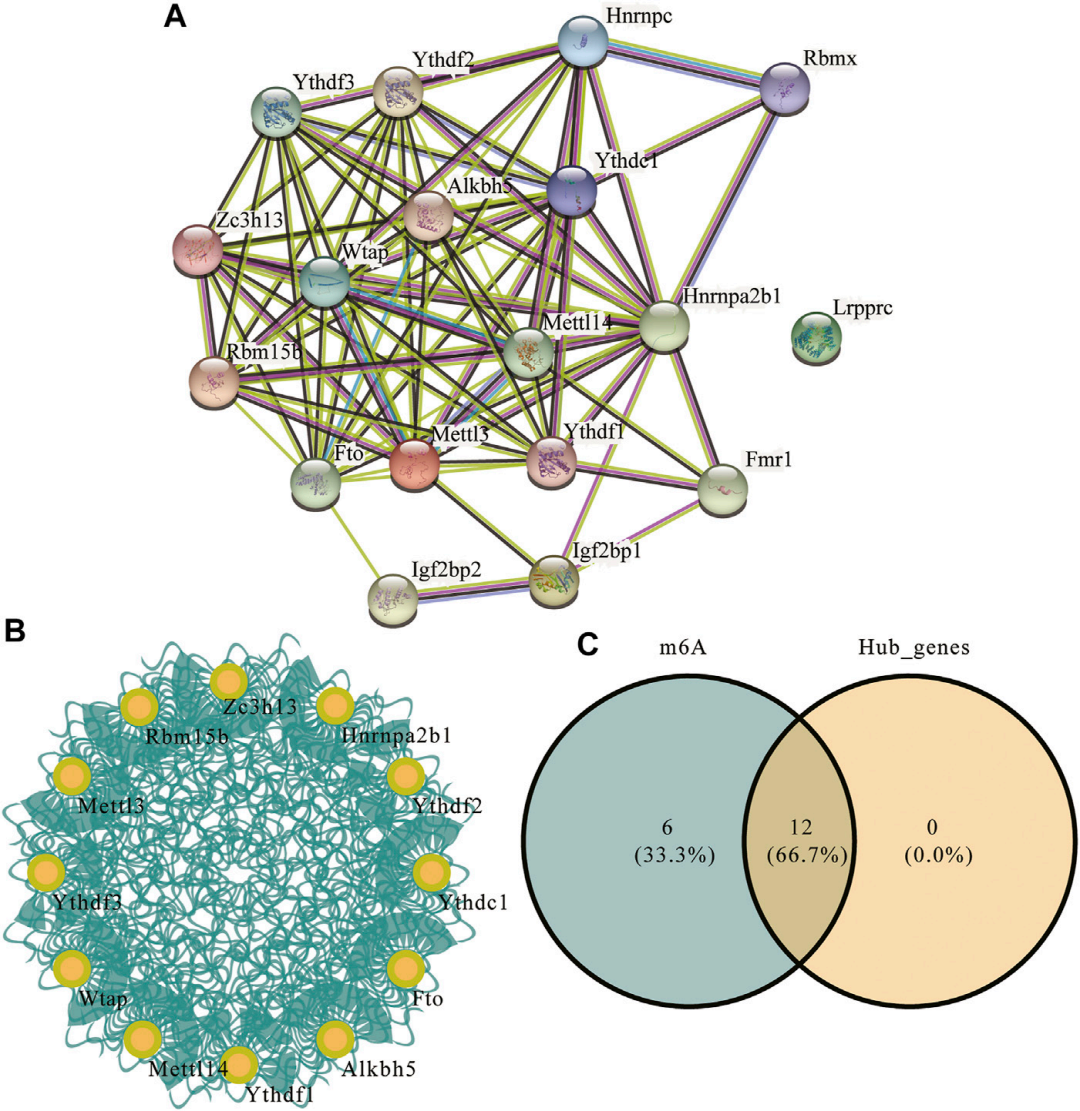
m6A regulator protein-protein interaction (PPI) network. **(A)** PPI network of the m6A regulators; the number of edges indicate the credibility of the evidence. **(B)** Network diagram of hub genes. **(C)** Venn diagram of hub genes and m6A regulators.

To explore the differences in PPI networks between the experimental and control groups, we extracted the protein interaction network of m6A methylation regulatory factors from each group. The effects of genes on biological signal transduction, gene expression regulation, energy and substance metabolism, cell cycle regulation, and other life processes were examined. We obtained the PPI of differentially expressed genes in the high- and low-risk groups using the STRING database (Figure 8A), which contained 83 interaction relationships and 18 m6A-related genes with an average local clustering coefficient of 0.719 and a PPI enrichment *p*-value of <1.0e^−16^. Functional interaction subnets were extracted using the MCODE plugin (Figure 8B). The results showed that the hub gene proportion of the m6A regulators in depression was 66.7% (Figure 8C).

### Immune Infiltration and Correlation in Experimental and Control Animal Models

**FIGURE 9:**
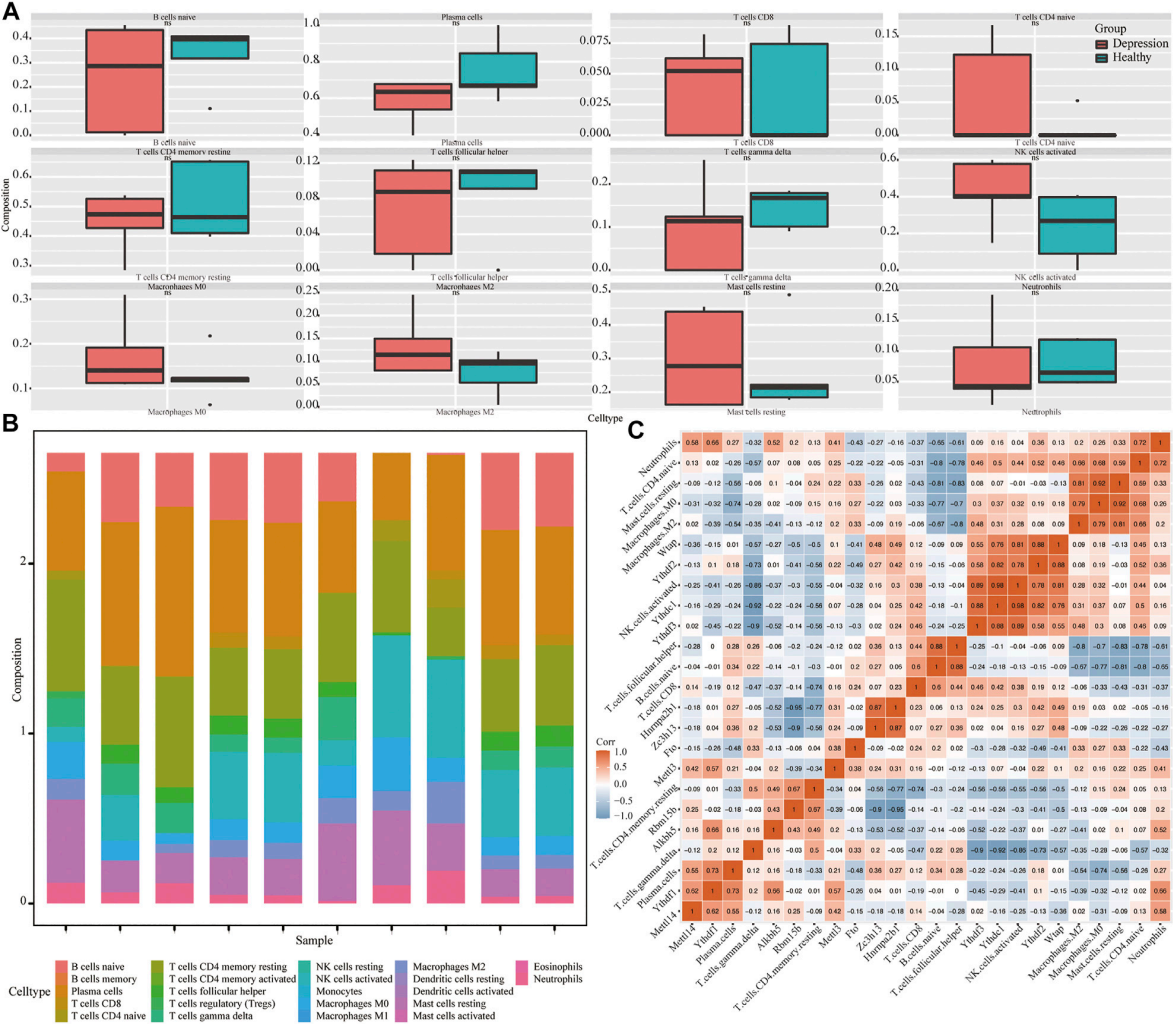
Analysis of immune infiltration in depression. **(A)** Differential expression of infiltrating immune cells in experimental and control groups. **(B)** Overall expression of infiltrating immune cells. **(C)** Correlation heat map of hub genes and immune-infiltrating cells.

Next, we assessed the overall immune profile and different levels of immune cell infiltration in the animal models of depression (Figures 9A,B). Compared with the control group, the contents of CD4 T cells in the experimental group were significantly increased. Additionally, the correlation between key genes of depression and immune infiltrating cells was significant (r > 0.7) (Figure 9C).

### Construction and Correlation Analysis of Relevant Molecular Subtypes of Depression

**FIGURE 10:**
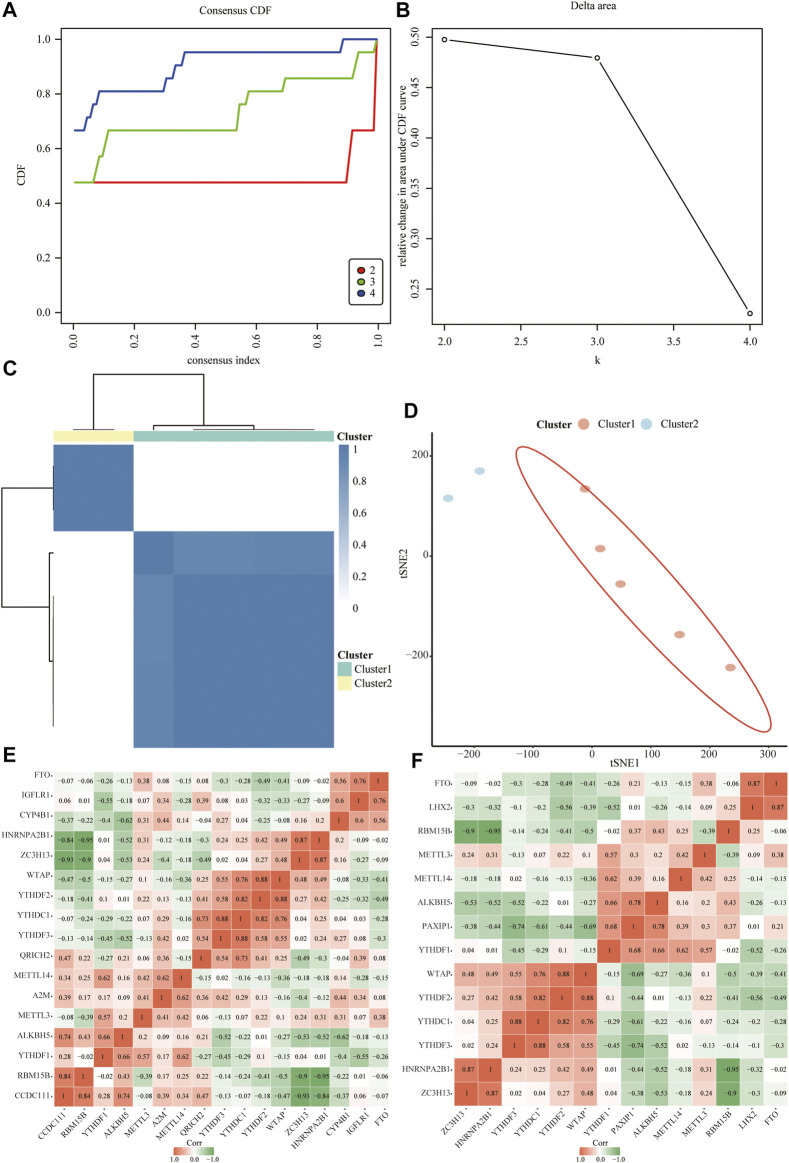
Relevant molecular subtypes and correlations of depression. **(A)** Cumulative distribution function (CDF) curve of consensus clustering of depression-related molecules; the abscissa is the consensus index and the ordinate is the CDF index. **(B)** Relative change in the area under the CDF curve; the results show that it is divided into two types, and the change trend is the most stable. **(C)** Cluster heat map of depression-associated molecular subtypes. **(D)** Principal coordinate analysis plot of depression-related molecular subtypes. **(E)** Heat map of the correlation between hub genes and isoform 1. **(F)** Heat map of the correlation between hub genes and isoform 2.

Next, we constructed relevant molecular subtypes of depression. According to the cumulative distribution function, the optimal number of subtypes was determined as two (Figures 10A–D). We then calculated the association of the hub genes with the two molecular subtypes of depression (Figures 10E,F).

## Discussion

Depression is a crippling and highly prevalent emotional disorder and the leading cause of suicide (Valverde et al., 2021). With an increase in life stress and social pressure, the prevalence and incidence of depression is increasing globally (Guo et al., 2020). The pathogenesis of depression remains unclear; a previous study showed that nearly 40% of patients administered antidepressants did not recover, and 20% of patients did not respond to any form of intervention (Brown et al., 2019). Epigenetic studies demonstrated a common genetic basis for psychiatric disorders, but the underlying molecular mechanisms remain largely unknown (Pineda-Cirera et al., 2022). Recently, m6A epigenetic modifications have gained attention. To determine the effect of m6A modification on the pathogenesis and immune infiltration of depression, we identified differentially expressed genes and successfully constructed a predictive model by comparing the experimental (depressed) group with the normal group. We found that the enriched modules and pathways were closely related to the immune response in depression, and 12 hub genes were identified in the PPI network. Analysis of immune infiltration showed that m6A modification is important in the differentiation of CD4+ T cells. Two molecular subtypes of depression were constructed, and the correlation between hub genes and two molecular subtypes of depression were analyzed.

Analyses of postmortem brain tissue from patients with depression showed that the pathology of depression involves abnormalities in specific brain regions, such as in the hippocampus (Campbell et al., 2004), pituitary (Fitzgerald et al., 2008), and cortex (Koenigs and Grafman, 2009). Therefore, the three datasets used in this experiment cover a wide range of tissues, including those of the hippocampus, pituitary, and cortex of depressed mice. We next performed de-batch effect and background correction of the GEO data to determine the location of the m6A regulators on chromosomes. Differential gene analysis of m6A regulators showed that *METTL3*, *FTO*, *YTHDF2*, *HNRNPC*, and three other genes were significantly upregulated in depressed rats, and correlation analysis was performed and the interaction relationship was described. As an m6A methyltransferase, *METTL3* plays an important role in epigenetics by promoting m6A methylation modification of RNAs (Liu et al., 2017). It has been reported that the expression of *METTL3* is significantly increased in patients with rheumatoid arthritis, affecting the secretion of inflammatory cytokines through the NF-κB pathway (Wang et al., 2019). *FTO* modulates the expression of targets such as ASB2 and RARA by reducing m6A levels in mRNA transcripts to enhance leukemic oncogene-mediated cellular transformation and leukemogenesis (Li Z. et al., 2017). A recent study showed that *FTO* modulates the expression of SIRT1 in the hippocampus by modifying adrenergic receptor β2 mRNA and affects the depression-like behavior of mice (Liu et al., 2021). Our results revealed similar evidence showing that m6a modification plays a key role in depression. *YTHDF2* can destabilize key gene transcripts and inhibit cancer cell proliferation (Sheng et al., 2020). Yu et al. (2019) found that the expression of *YTHDF2* in macrophages was upregulated after lipopolysaccharide stimulation and that knocking out *YTHDF2* reduced the levels of pro-inflammatory factors. *HNRNPC* plays an important role in various cancers and neurodegenerative diseases such as Alzheimer’s disease (Geuens et al., 2016). Studies have suggested that *HNRNPC* dysfunction causes Parkinson’s disease through immune inflammation (Quan et al., 2021).

In the PPI network identified in this study, the hub gene proportion of the m6A regulator in depression was 66.7%, confirming it profound impact on the occurrence and development of depression. By constructing a clinical prediction model, the effect of the m6A regulator may further explain its application value in the diagnosis of depression. In analysis of the functional regulatory effect of m6A on RNA, m6A-related regulatory factors were enriched in the spliceosome KEGG pathway, which plays an important role in the pathogenesis of various autoimmune diseases (Verma et al., 2018). The results of GO analysis, GSEA, and GSVA showed that depression is closely related to the regulation of inflammatory gene expression and infectious disease pathways. Our findings support the pivotal role of inflammatory responses in the regulatory network of depression (Woo et al., 2018).

In immune infiltration that, compared to in the control group, the content of CD4 T cells in the experimental group was significantly increased. This result is consistent with the experimental findings of Li H.-B. et al. (2017), indicating that m6A methylation plays an important role in CD4 T cell differentiation. Recent findings suggested that *METTL3* promotes M1 macrophage polarization and possesses pro-inflammatory effects (Liu et al., 2019). *FTO* may affect the release of inflammatory cytokines and reduce the risk of inflammatory diseases (Cheng et al., 2021). The role of *YTHDF2* in preventing excessive inflammatory responses is a potential therapeutic target for inflammatory diseases (Yu et al., 2019). One study classified severe asthma into three m6A modification patterns, which can achieve precise treatment according to the characteristics of the immune microenvironment (Sun et al., 2021). Lynall et al. (2020) stratified patients with depression according to their leukocyte subsets and found that the inflammatory depression subgroup had more severe depressive symptoms. Therefore, we constructed two molecular subtypes of depression and calculated the correlation between the hub genes and two molecular subtypes of depression. The immune characteristics of different models can provide a theoretical basis for classifying the immune subtypes of depression. Clinical trials have focused on the efficacy of drugs targeting inflammatory molecules in patients with depression. For example, a meta-analysis confirmed the role of celecoxib, a nonsteroidal anti-inflammatory drug that inhibits prostaglandin synthesis, in enhancing antidepressant efficacy (Na et al., 2014). Given the relationship between inflammatory cytokines and treatment resistance, a clinical trial was conducted to test the tumor necrosis factor antagonist infliximab in patients with treatment-resistant depression (Raison et al., 2013). As patients with depression have different inflammatory states, inflammation-based stratification may help optimize the antidepressant effects of anti-inflammatory drugs (Köhler-Forsberg et al., 2019).

Currently, epigenetic studies in the field of depression are limited and varied. Research on the m6A modification mechanism and immune microenvironment theory to explore the pathogenesis and treatment direction of depression is can make up for the gap in epigenetic modification and inflammatory infiltration in depression. However, this study had some limitations. First, this study was based on bioinformatics analysis, and the results require verification in animal and human experiments and clinical settings. Second, the tissue sample size collected was small, particularly the gene chips of depressed rats. Although the clinical prediction model constructed in this study showed a high degree of agreement (test and validation set area under the receiver operating characteristic curves: 0.855 and 0.75, respectively), the detection ability of the model must be improved by integrating multiple volume data in further studies. Finally, because of the heterogeneity of depression and lack of clinical data, not all patients with depression have obvious inflammatory infiltration, and additional inflammatory response characteristics of patients with depression must be included in further subgroup analyses.

We identified 12 candidate genes as potential diagnostic biomarkers through PPI network analysis and functional enrichment and further explored the m6A regulatory factor in the inflammatory infiltration of depression and inflammation-based stratification studies. Our findings confirm the positive effect of m6A modification on the immune properties of depression and provide insight into the pathogenesis and treatment of depression.

## Data Availability

The original contributions presented in the study are included in the article/Supplementary Material, further inquiries can be directed to the corresponding author.
